# Deletion of Crry, the murine ortholog of the sporadic Alzheimer's disease risk gene CR1, impacts tau phosphorylation and brain CFH

**DOI:** 10.1016/j.neulet.2012.11.008

**Published:** 2013-01-15

**Authors:** R. Killick, T.R. Hughes, B.P. Morgan, S. Lovestone

**Affiliations:** a King's College London, Institute of Psychiatry, De Crespigny Park, Denmark Hill, London SE5 8AF, UK; b Institute of Infection and Immunity, School of Medicine, Cardiff University, Cardiff CF14 4XN, UK

**Keywords:** Alzheimer's, Tau, CR1, Crry, CFH, Sporadic

## Abstract

Large-scale genome-wide SNP association studies have identified an association between variants of CR1, the gene encoding complement component receptor 1, and the sporadic form of Alzheimer's disease. The role of CR1 and the complement system in Alzheimer's disease remains far from clear. In rodents the closest ortholog of CR1 is the Crry gene (Cr1-related protein Y). To begin to explore its role in Alzheimer's disease we examined hippocampal lysates from Crry^−/−^ mice and age matched controls by immunoblotting. We measured complement factor H, a component of the complement system and biomarker for Alzheimer's disease progression, and tau phosphorylation at the serine 235 site, hyperphosphorylated forms of tau being a defining neuropathological hallmark of the disease. We found that levels of CFH and of tau phosphorylation at serine 235 were strongly and significantly reduced in Crry^−/−^ samples. These observations provide a starting point for further attempts to determine the role of CR1 in the neuropathological process driving Alzheimer's disease.

## Highlights

► Crry is the murine ortholog of the sporadic Alzheimer's risk gene CR1. ► Deletion of Crry results in decreased tau phosphorylation. ► Deletion of Crry results in reduction in brain CFH – a biomarker of disease progression.

## 1 Introduction

Up to 5% of Alzheimer's disease (AD) is familial in origin, arising from single mutations in one of three genes, APP, PSEN1 and PSEN2. This familial form of the disease is aggressive, with an early age of onset. The identification of the familial genes has helped to formulate, and then strengthen, the amyloid cascade hypothesis of AD aetiopathology, which holds that it is an increase in the generation (or a reduction in the clearance) of β-amyloid, or a relative increase in the more amyloidogenic forms of β-amyloid in brain, that initiates the disease process, resulting in the aberrant phosphorylation of tau and its intracellular aggregation in the form of neurofibrillary tangles (NFTs) [13]. β-Amyloid also forms aggregates, extracellularly in the form of senile plaques: Plaques and tangles being the defining neuropathological hallmarks of the AD brain. However, any detailed description of the mechanism driving this hypothetical cascade at the biochemical/molecular level remains lacking [12].

The vast majority of AD is not familial but sporadic with an age of onset of 65 years and above. The late onset form of the disease (LOAD) also has a genetic component but one that is more poorly defined [8]. For almost 20 years the only known risk factor for LOAD was the ɛ4 allele of APOE [32] and the role of apoE in AD pathology also remains undetermined [6]. Recently, a number of very large genome wide SNP association studies identified several new loci carrying risk for sporadic AD [14,15,21], although each to a lesser extent than does APOE. Among these loci (possibly up to nine to date, accounting for up to 50% of LOAD genetics [24]) are; CLU, the gene encoding the secreted protein clusterin which can protect cells against complement induced cytolysis; and CR1, encoding Complement receptor 1, a complement regulator and receptor for complement component C3b.

Using large-scale ‘un-biased’ proteomic approaches we have identified clusterin and another complement component, complement factor H (CFH), as blood based biomarkers for AD [16,35]. We find that Clusterin levels are associated with atrophy of the entorhinal cortex, disease severity, and rate of progression [35] and that CFH plasma levels show a significant positive correlation with measures of cognitive decline [34]. In addition CFH protein levels have also been reported to be raised in AD brain [33]. Together, these observations indicate that the complement system likely plays an important role in the mechanism underlying AD pathogenesis.

In man CR1 and CR2 are encoded by separate genes, whilst in mice they are generated from alternatively spliced transcripts from the Cr2 gene [10,19,20,22,28]. Although murine CR1 and CR2 proteins bind directly to C3b [26] and form rosettes with C3b-coated particles [18,23], they are not found on the surface of platelets or unstimulated neutrophils [18]. Thus neither CR1 nor CR2 are the functional murine C3b receptor of platelets and neutrophils. Mice carry a rodent specific gene, Cr1-related protein Y (Crry), an important cell-surface regulator of complement that bears greater similarity to human Cr1 in terms of protein sequence and function than does murine CR1. Sequence homology demonstrates that the human CR1 gene has evolved from the Crry gene. This conclusion is supported by the relative locations of the Crry/CR1 genes with respect to the Cr2/CR2 genes in mouse and man. Given this relationship we examined brain tissues from Crry^−/−^ mice in order to investigate what role CR1 might play in AD, using Crry deletion as a murine model of human CR1 gene deletion.

## 2 Methods

Deletion of Crry is embryonically lethal as maternal complement destroys the unprotected embryo [37]. We deleted murine Crry and rescued Crry-deficient embryos by back-crossing onto a C3 deficiency; however, the resultant mice have no functioning complement system. To generate Crry-deficient mice with a normal complement system, heterozygous Crry-deficient mice were crossed and the dam treated with a blocking anti-C5 mAb through pregnancy [30]. Healthy Crry-deficient pups were obtained and interbred to build a colony. These mice displayed no obvious phenotype but testing revealed chronic complement activation and consumption of C3. As we previously reported microscopic examination of brain and spinal cord showed abundant microglial priming, which is required for complement activation in the central nervous system [29].

To examine CFH and tau in hippocampi we collected brains from 20 week old Crry^−/−^ mice and age matched WT controls (*n* = 10/group). These animals have a normal life span and we chose to examine hippocampi at this age, i.e., young but fully mature adults, to avoid any developmental effects on tau phosphorylation levels. Brains were snap frozen and the hippocampus subsequently removed whilst at 4 °C, homogenised, protein extracts prepared and subjected to immunoblotting as previously described [17].

## 3 Results

**Fig. 1:**
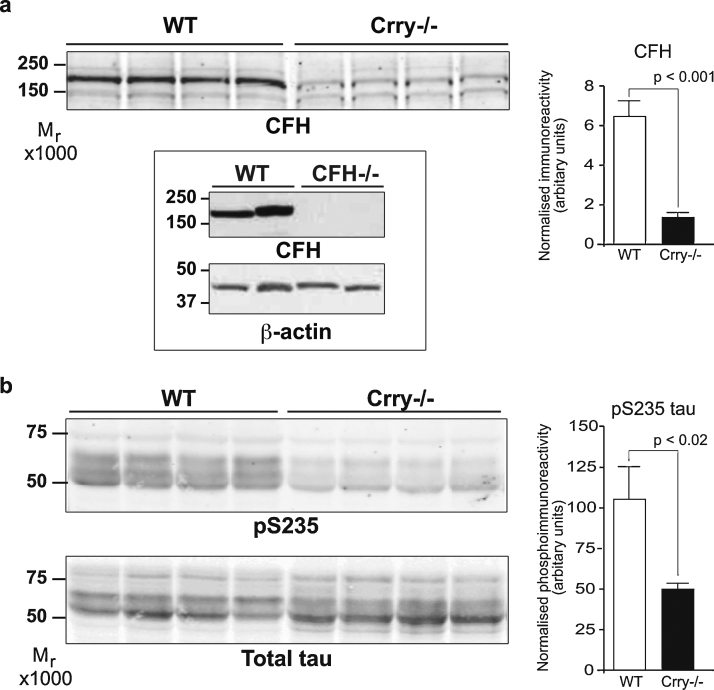
Crry deletion reduces CFH and tau phosphorylation. (a) Immunoblot of hippocampal protein lysates probed for CFH, left hand panel. Densitometry was performed and is shown in bar graph, right. Specificity of the CFH antibody is shown on wild type and CFH knockout brain tissue, inset panel. The apparent molecular weight of the CFH immunoreactive band is between ∼165 kDa. (b) Immunoblots showing MC6 immunoreactivity, top panel, and total tau immunoreactivity using DAKO A0024, bottom panel. Densitometry values obtained for MC6 were normalised to total tau protein values and are presented in the bar graph, right. Significance values were determined by Student's *T*-test. *n* = 10 per group. Error bars = SEM.

Using the well-characterised CFH polyclonal antibody A312 (Quidel, San Diego, CA, USA) for immunoblotting a large and highly significant reduction in CFH was observed in Crry^−/−^ hippocampus compared to controls (Fig. 1a). In all other tissues examined, including liver, CFH levels were not significantly different in the Crry^−/−^ animals from those of controls (data not shown).

Next we examined tau phosphorylation in the same hippocampal lysates. Three phosphorylation sites in tau, threonine 231, serine 235 and serine 262, are key regulators of its binding to microtubules [3,7,31] and when occupied enhance fibril formation [1,2,5,25]. We examined the phosphorylation state of tau using the phospho-specific antibody MC6 that recognises phospho-serine residue 235 (pSer235 – a generous gift from Peter Davies, Albert Einstein College of Medicine, USA). pSer235 is also part of the phosphoepitope, AT180, immunoreactivity to which is greatly increased in AD brain and is used in the postmortem diagnosis of the disease [11,27]. A marked and significant reduction in MC6 immunoreactivity in Crry^−/−^ samples compared to controls was observed (Fig. 1b upper panel).

Tau exists as multiple splice forms, manifesting as multiple bands on protein gels/western blots. Of note, when using the “total tau” antibody A0024 (Dako UK Ltd., Ely, UK) to normalise MC6 phospho-immunoreactivity levels to, we observed that while total hippocampal tau protein levels are not significantly different between Crry^−/−^ mice and controls of the two major tau species observed the slower migrating one was generally less intense, and the faster migrating one, generally more intense, in Crry^−/−^ samples. This faster electrophorectic migration of tau in Crry^−/−^ samples may reflect further decreases in tau phosphorylation at other phosphoepitopes (Fig. 1b lower panel).

## 4 Discussion

The identification of CR1 by GWAS strongly implicates a role for it and the complement system in AD pathogenesis. Our data show that deletion of Crry, the murine orthologue of CR1, gives rise to reduced hippocampal levels of CFH and decreased phosphorylation of tau at one of the key phospho-residues controlling its microtubule binding, serine 235. Given that plasma CFH is a biomarker for AD progression and hyperphosphorylated tau is the major constituent of neurofibrillary tangles, a defining hallmark of the disease, these observation support the idea that CR1 plays a role in the disease process, although the mechanism by which loss of Crry gives rise to either of these affects or if the two are connected has still to be determined.

Complement component proteins are intimately associated with β-amyloid plaques [9] and Aβ peptides can activate complement leading to the generation of C3b [36], which then needs to be cleared from brain. As the longer forms of CR1 contain more C3b binding sites and facilitate more rapid clearance than do the shorter forms, it has been suggested that longer forms of CR1 might confer relative protection by dampening complement activity, based on the assumption complement activity is pathogenic [8]. However, Brouwers et al. [4] have shown that it is the longer forms of CR1 that impart risk for AD, suggesting complement activation is neuroprotective and that it is increased inhibition of complement activity by longer CR1 alleles that confers risk.

Our data indicate that complete loss of Crry, and by extrapolation CR1, leads to a decrease in tau phosphorylation at serine 235 and a reduction in CFH. As these are both markers of AD pathology, it indicates that CR1 activity is a part of the disease process, driving tau phosphorylation and CFH production, and that risk alleles must confer a pathogenic gain in activity. Further elucidation of the role of Crry/CR1 in brain will doubtless contribute to our better understanding of the Alzheimer's disease process.
