# Corrigendum to “Deletion of Crry, the murine ortholog of the sporadic Alzheimer's disease risk gene CR1, impacts tau phosphorylation and brain CFH” [Neurosci. Lett. 533 (2013) 96–99]

**DOI:** 10.1016/j.neulet.2013.05.004

**Published:** 2013-06-17

**Authors:** R. Killick, T.R. Hughes, B.P. Morgan, S. Lovestone

**Affiliations:** a King's College London, Institute of Psychiatry, De Crespigny Park, Denmark Hill, London SE5 8AF, UK; b Institute of Infection and Immunity, School of Medicine, Cardiff University, Cardiff CF14 4XN, UK

In the methods section and in the figure legend for Fig. 1, the *n* per group was incorrectly stated as 10 per group. The correct *n* for each group is WT, *n* = 7; Crry^−/−^, *n* = 10.

